# Decoding Neurodevelopment: Findings on Environmental Exposures and Synaptic Plasticity

**DOI:** 10.1289/ehp.120-a70

**Published:** 2012-02-01

**Authors:** Angela Spivey

**Affiliations:** Angela Spivey writes from North Carolina about medicine, environmental health, and personal finance.


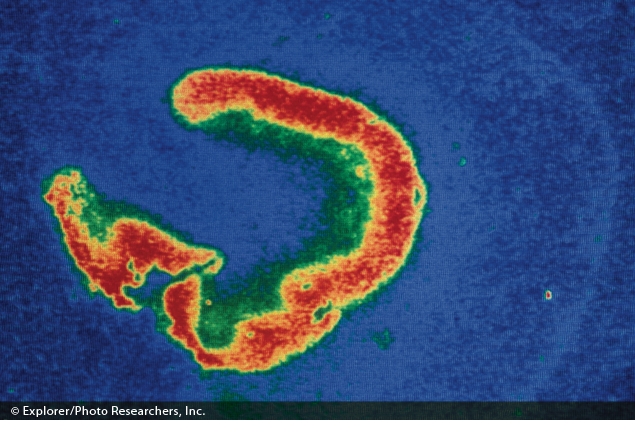
What makes one person different from the next? In large part it’s that our individual brains are wired a bit differently; each neuron in each person’s brain has a different set of synapses connecting to a different set of neurons. When we’re born, most of the major functional subregions of the brain and their interconnections are in place, but various experiences and environmental exposures affect which synaptic connections become stronger and which become weaker. Different areas of the brain have this synaptic plasticity—or ability to grow stronger or weaker connections—at different time periods, and the timing of these “critical periods” is tightly regulated.

Thermogram showing hormone receptors in the hippocampus of an infant. Exposures to certain neurotoxic agents during critical periods of synaptic plasticity may set the stage for developmental disorders.

© Explorer/Photo Researchers, Inc.

The field of neurobiology is humming with new findings on the intricate workings that orchestrate this process, too many to describe in one story. But of special interest are key discoveries by investigators at the National Institute of Environmental Health Sciences (NIEHS) about some of the basic mechanisms involved in synaptic plasticity, and work by other investigators that explores the hypothesis that environmental toxicants that disrupt synaptic plasticity at critical periods play a role in disorders that have roots in early brain development, such as autism spectrum disorders (ASDs), attention deficit/hyperactivity disorder, and schizophrenia.^1^^,^^2^

## The Conspiracy against Plasticity in CA2

All areas of the brain were once thought equally capable of rewiring themselves in response to environmental input, but Serena Dudek, principal investigator of the NIEHS Synaptic and Developmental Plasticity Group, has revealed that one region of the brain has what she and colleagues have termed a “conspiracy against plasticity,” with the neurons there exhibiting a remarkable stability in synaptic strength in response to electrical stimulation.^3^ This region, CA2, is part of the hippocampus, an important center of learning and memory. The other regions of the hippocampus, CA1 and CA3, have been heavily studied, but CA2 had been largely ignored; it was long considered simply a transition area between the other regions.^3^^,^^4^

Dudek began studying CA2 because she wanted to figure out exactly how critical periods for phenomena such as ocular dominance are controlled. Ocular dominance refers to the preference of the brain for input from one eye over the other, and development of ocular dominance provides a simple example of synaptic plasticity at work in everyday life. When a baby is born with a cataract or other condition in only one eye, the problem ideally should be corrected before 2 years of age and no later than age 8.^5^ That’s because the longer the brain receives information from only one eye, the weaker its connections to the inactive eye will become, until that eye is wired out of the circuit, even if the physical condition itself is later corrected.^6^ But if an adult loses vision in one eye for a time, the consequences are not so dire; the connections to receive information from both eyes are already formed, and the opportunity for that part of the brain to rewire itself has closed.

In rodent models Dudek noticed that a particular gene, TREK-1, was highly expressed in a layer of the adult visual cortex that wasn’t particularly plastic, and that its expression appeared to increase as the animal aged and the critical period for plasticity in that region closed.^7^ “This gene was one of several that might have been predicted to prevent plasticity, and so we were surprised to see that it showed up in this area of the hippocampus, CA2.^8^ As we studied CA2, it turned out that the area was a lot different [from CA1 and CA3],” Dudek says.

**Figure:**
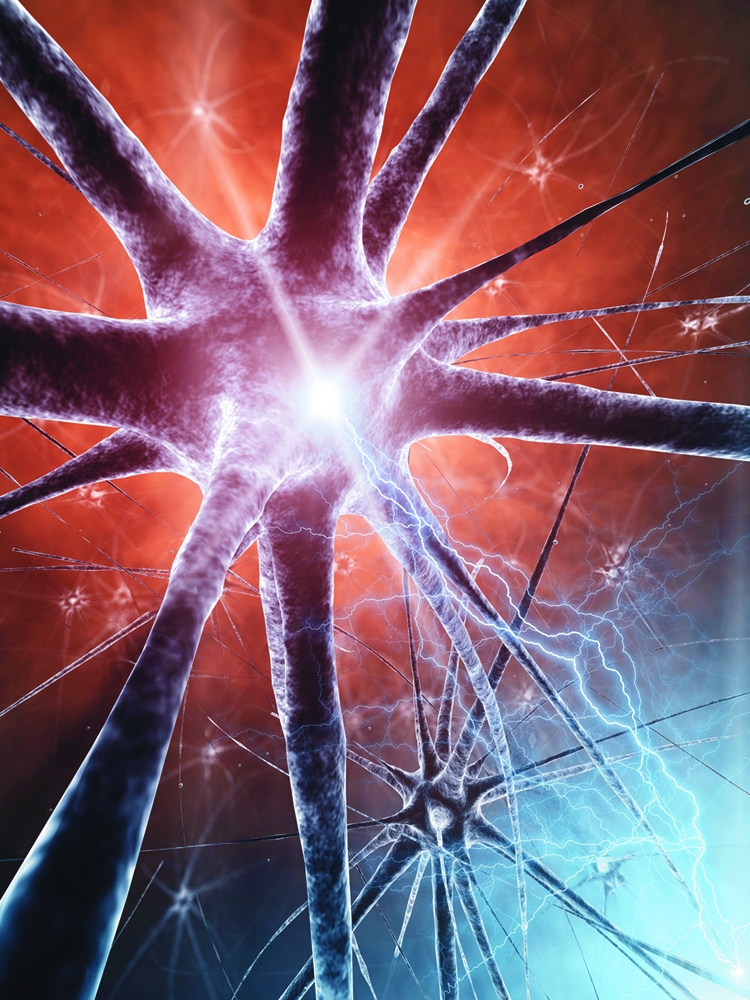
The Basics of Neural Activity Neurons are polarized brain cells: that is, the outside of the cell membrane is positively charged, while the inside is negatively charged. On each neuron a large antenna-like tree of dendrites receives synapses from many other neurons, and a single axon branches to contact many other neurons. Neurons communicate with each other using chemical signals that they release at synapses, where the axon terminals contact another neuron’s dendrites. Some chemicals alter the activity of ion channels, while others alter neuronal metabolism. When enough excitatory synapses are activated simultaneously, the neuron conducts a regenerative electrical wave of depolarization along its axon. This electrical wave is known as an “action potential,” sometimes referred to as the “firing” of a neuron. When the depolarization arrives at the axon terminals, or synapses, the resulting calcium entry triggers the release of vesicles containing chemical messengers called neurotransmitters, and the process begins again. Synaptic plasticity is the ability of the synapses to strengthen or weaken their connections. A simple way to think of strengthening a synapse is the insertion of postsynaptic receptors. When the number of receptors is increased, neurotransmitters can have more effect. When postsynaptic receptors are removed, neurotransmitters make less of an impact per transmission. Veer

In their studies of CA2, Dudek and colleagues used the patch-clamp technique to measure the response of neurons in slices of rodent hippocampus to electrical stimulation; these responses include either the firing of “action potentials” or else the smaller electrical activity of synapses [see “The Basics of Neural Activity,” this page]. Repeated high-frequency electrical stimulation to other regions of the hippocampus typically causes a long-lasting boost in the strength of synapse; this is called long-term potentiation. But stimulating slices from CA2 yielded no such increase.^3^

“Up until we discovered this five years ago, I would have thought that the cellular profile of all the pyramidal [primary excitatory] cells in the cerebral cortex was similar enough that their synaptic plasticity would be very similar. But we’re finding that that is clearly not the case,” Dudek says. Neuroscientists are excited about her findings in CA2 because they open up new opportunities for delving into the workings of plasticity, especially for better understanding the factors that reduce or prevent it.

## Bidirectional Activity

In earlier work, when Dudek was a PhD student, she conducted the first studies demonstrating that synaptic plasticity goes in both directions. Many neuroscientists had shown that long-term potentiation occurs, but no one had shown that the opposite—long-term depression—also could be induced. Dudek and colleagues demonstrated that a repeated stimulation of synapses at frequencies lower than those required to induce long-term potentiation would result in prolonged synaptic weakening in hippocampal slices.^9^

Later, Dudek collaborated with John Hepler, a professor of pharmacology at Emory University, to find that RGS14, a gene highly enriched in CA2, keeps plasticity turned off. When Hepler knocked out RGS14 in rodents, the animals learned faster, especially in spatial activities, and Dudek showed that plasticity had been turned back on in CA2.^10^ It may seem odd that the brain has a “Homer Simpson gene,” as RGS14 has been nicknamed, that keeps the brain from learning. But Hepler suggests that such mechanisms may be vital for processing the world in a way that enables normal function.

“Think about everything that you encounter every day: You forget the vast majority of it, and that’s a good thing—you have to filter out what’s important and what’s not important,” Hepler says. “You could also think about RGS14 and CA2 as important devices for filtering which memories are important to retain and which are not.”

Most recently, Dudek and colleagues have found that CA2 is probably the “caffeine center” of the brain. In both brain slices and live animals, they demonstrated that a dose of caffeine equivalent to about 2 cups of coffee for a human almost doubled the transmissions of synapses in CA2 compared with those in controls.^11^ At the same doses, caffeine had no effect on other areas of the hippocampus. Dudek thinks that CA2 gets such a jolt from caffeine because the region is rich in adenosine receptors. Adenosine is a neurotransmitter that normally inhibits synaptic transmission and plasticity; that is, it induces sleepiness. Caffeine is known to antagonize adenosine receptors.

## The Hormone Connection

Some of the signaling chemicals involved in plasticity are hormones. For instance, since the early twentieth century, it has been known that people deprived of iodine develop cognitive disabilities because the lack of iodine reduces production of thyroid hormone.^12^ But many of the details of how thyroid hormone actually works in the brain are still largely unknown.

Researchers previously thought that thyroid hormone affected neurodevelopment mainly by regulating gene expression, a process that can take hours to produce effects. But in recent years, says David Armstrong, chief of the NIEHS Neurobiology Laboratory, it has become apparent that thyroid hormone also works via rapid events, such as binding to receptors that can regulate ion channel activity through cytoplasmic signaling mechanisms.

Armstrong and colleagues are finding out how thyroid hormone does its job in normal brain development. In a rat pituitary cell line, they showed that a nuclear receptor for thyroid hormone, TRβ, binds to phosphatidylinositol 3-kinase (PI3K), an enzyme that’s essential for brain development and synaptic plasticity.^13^ In unpublished work presented in November 2011 at the Society for Neuroscience Annual Meeting, Armstrong’s team showed exactly where TRβ binds to PI3K and that, when a mutation at this site prevents binding, rats’ brains exhibited less plasticity, reduced synaptic response to an electrical stimulus, and reduced response to thyroid hormone.^14^

“If we change one tyrosine residue in the thyroid hormone receptor protein to a phenylalanine, which is a very conservative difference, then we see all these changes in the brain,” Armstrong says. “So this must be an important mechanism for how thyroid hormone works.”

Tracing these intricate basic mechanisms is the best route that Armstrong sees to the discovery of environmental toxicants that may disrupt brain development. Other approaches that involve exposing an animal to a suspected toxicant and observing behavioral changes that develop are too imprecise, in his view. “Especially for things like early learning disorders in humans, it’s not so easy to map that [type of behavioral change] onto mouse behavior,” he explains. “It’s highly controversial what an autistic mouse would look like because they have a very different forebrain and cortex than we do and very different social behaviors.”

Armstrong and other basic scientists therefore take the approach of nailing down the molecular mechanisms that underlie brain functions such as plasticity. Then they can develop high-throughput screens to identify toxicants that may perturb those mechanisms.

## Too Much Excitement?

How might a disruption in synaptic plasticity lead to developmental disorders such as ASDs? In order for senses such as vision to develop normally and become integrated into behavior, the brain changes in response to experience, says Michela Fagiolini, a neurobiologist at Harvard Medical School. Some evidence suggests that ASDs—a group of conditions of varying severity—begin in part with an underdeveloped ability to integrate visual information with auditory information when making out words. Other research has shown that autistic children process images of faces differently than nonautistic children. In one study, autistic children were more successful at recognizing facial expressions depicted with high spatial frequency; that is, their visual perception was more oriented to contrast and detail than that of nonautistic children, who focused on the overall composition of the faces.^15^

Normal plasticity requires a delicate balance between excitation and inhibition of synapses, according to Fagiolini. “We and others have shown that a balance between inhibition and excitation is actually critical for allowing the brain to become plastic and respond to the environment,” she says. “This is different from what people thought in the past, when inhibition was thought to suppress plasticity. The new view is that you need a certain level of inhibition to allow the brain to sense and respond to the environment.”

In one study, scientists genetically engineered mice to lack just 1 of 2 receptors for γ-aminobutyric acid (GABA), a neurotransmitter involved in inhibition of synaptic electrical excitement.^16^ Without the proper inhibition, the visual cortex stayed in the immature state that normally occurs just before the onset of the critical period for ocular dominance. But enhancing GABA transmission with the drug benzodiazepine resulted in a normal-length critical period of plasticity, no matter the mouse’s age.

Some scientists hypothesize that lack of inhibition of electrical activity in the brain caused by environmental toxicants may contribute to developmental disorders such as ASDs. For instance, Timothy syndrome, a very rare developmental disorder characterized by autism and cardiovascular irregularities, results from a mutation in the Ca_V_1.2 voltage-gated calcium channel.^17^ Voltage-gated ion channels open in response to electrical stimulation to allow entry of critical signaling ions including calcium, potassium, and sodium. Armstrong and colleagues found that this Ca_V_1.2 mutation increases the duration of each channel opening by more than 10 times, and far more calcium enters the cells to the point it could become cytotoxic.^18^

Increased calcium entering through Ca_V_1.2 channels also overstimulates any nearby ryanodine receptor calcium channels, which are thought to work with Ca_V_1.2 to control synaptic plasticity as well as muscle coordination.^19^^,^^20^ “Normally, ryanodine receptors are kept under tight control,” says Isaac Pessah, chairman of the Department of Molecular Biosciences at the University of California, Davis. “For instance, in muscle cells such as those in the heart, ryanodine receptors’ role is to contribute an intense signal only when they’re called upon. When muscle contraction is needed, the receptors open briefly, release the calcium needed, then shut off quickly.” But some polybrominated diphenyl ethers (PBDEs) and polychlorinated biphenyl ethers (PCBs), because of their structure, enhance the activity of ryanodine receptors.^19^^,^^21^

Pessah’s group has shown that after exposure to PCBs, ryanodine receptors activate much more easily than usual, and they are, as Pessah says, “noisy.”^19^ “I call it ‘chattering,’” he says. “That can be extremely confusing to cells.” Pessah and colleague Pam Lein, also at UC Davis, have shown that some of the same PBDEs and PCBs that alter the activity of ryanodine receptors also affect activity-dependent growth of dendrites (the branches of neurons where synaptic receptors lie).^22^

“Activity-dependent growth is extremely important in neurodevelopment because not only does it develop the complexity needed to promote learning, it also is essential for ‘coding’ neural networks so that they’re meaningful to the brain,” Pessah says. He and Lein hypothesize that the effects of the environmental toxicants on neural development are mediated by their effects on ryanodine receptors in neurons, but that idea remains to be tested in animals.

## What Does This Mean for Humans?

More than 150 mutations in one type of ryanodine receptor (Ry1R1) have been well documented to heighten sensitivity to chemicals called halogenated alkanes, which are used as general anesthetics. A person may go his whole life unaware that he has such a mutation until he is given a halogenated anesthetic in the operating room, which could rapidly trigger a life-threatening response known as malignant hyperthermia.^23^^,^^24^^,^^25^

“We are convinced that this is an amazing demonstration of a gene-by-environment interaction in which the gene by itself is insufficient to cause an overt clinical phenotype, so you don’t know that individuals are susceptible until they’re subjected to an environmental stressor,” Pessah says. He and other researchers are investigating the idea that ASDs and other developmental disorders similarly could begin with a small genetic variation that makes a person more susceptible to ryanodine receptor disruption.

Can human developmental disorders really be reduced to disruptions in synapses and chemicals? The idea may not be so farfetched. As Armstrong points out, until it was demonstrated a few years ago, scientists would not have predicted that intranasal administration of the hormone oxytocin, previously thought to be involved mostly in reproduction, could have a direct effect on complex human behavior such as trust.^26^

Because many of the mechanisms of neural plasticity are chemical processes, investigators predict that environmental chemicals are capable of disrupting them. However, although the molecules that regulate synaptic plasticity in mice and humans are almost identical, the cellular substrates of cognitive processes in the two species are quite different. So it remains to be seen what any of these findings mean for human neurodevelopment.

But even if basic work such as Dudek’s and Armstrong’s can pinpoint particular chemicals that disrupt plasticity in experiments at high concentrations, many steps will be required to determine the human health effects of environmentally relevant chemical exposures. “There won’t ever be any one test that rules a chemical in or out as being neurotoxic,” Armstrong says. “You have to test its toxicity at many levels.”
